# Evidence of sweet corn yield losses from rising temperatures

**DOI:** 10.1038/s41598-022-23237-2

**Published:** 2022-10-29

**Authors:** Daljeet S. Dhaliwal, Martin M. Williams

**Affiliations:** 1 Department of Crop Sciences, University of Illinois at Urbana Champaign, Urbana, IL USA; 2 Global Change and Photosynthesis Research Unit, USDA-ARS, Urbana, IL USA

**Keywords:** Ecology, Agroecology

## Abstract

Crop production is sensitive to anomalous weather conditions, but vegetable crops can be highly sensitive to environmental changes. Using sweet corn data collected on 16,040 fields over a 27-year period, we: (a) estimate yield sensitivities to changes in growing season temperature and total precipitation, (b) estimate critical thresholds in non-linear temperature effects on sweet corn yield across diverse environments, and (c) quantify yield losses from surpassing the upper temperature threshold during anthesis in sweet corn. Our results show growing-season temperatures exceeding 30 $^\circ{\rm C}$ were detrimental to crop yield. Each additional degree day spent above 30 $^\circ{\rm C}$ during anthesis reduced crop yields by 0.5% and 2% in irrigated and rainfed fields, respectively. This study shows evidence for sweet corn yield losses across broad spatial domains in the wake of climate change and underscores the urgency to accelerate crop adaptation strategies to sustain production of this highly popular crop.

## Introduction

Climate change is likely to affect global, regional, and local food security^1^. Recent years have revealed an increase in frequency and intensity of extreme weather events—including heatwaves, droughts, floods—that will worsen as global temperatures continue to rise from increasing greenhouse gas concentrations^2^. Over the last fifty years, extreme weather events related to climate change have cost USD 3.64 trillion worldwide^3^. Last year the U.S. alone suffered losses exceeding USD 1 billion from climate- and weather-related disasters^4^. With evidence accumulating that global climate change is occurring at a rate faster than predicted, development and adoption of crop adaptation strategies is critical.

Rapidly increasing human population coupled with the ubiquitous effects of climate change, which include rising temperatures and shifting precipitation patterns, put an incredible strain on our agricultural systems. There is a general consensus that future climate scenarios, characterized by an increase in the intensity and frequency of extreme weather events in the U.S. grain belt, will result in yield losses for staple cereal crops across the globe^5–9^. The impact of climate change on vegetable crops, which are often more sensitive to environmental conditions^10,11^, are more nutrient-dense^12^, and generate more employment than cereal crops^13^, have received comparatively little study.

A wealth of research shows crop yield losses from abiotic stress are greatest in the reproductive period^14–17^. For instance, by the end of the century, global field corn yield losses are expected to double due to extreme heat stress at anthesis^18^. Yield losses in field corn due to heat stress at anthesis have been linked to kernel abortion resulting from reduced sink potential, developmental asynchrony, ovule abortion, and pollen sterility^19–22^. However, a comprehensive evaluation of the implications of temperature-induced stress at anthesis in sweet corn—a popular vegetable that differs greatly from field corn germplasm and agronomy—is lacking.

The U.S. is a major player in the global food market and drives agricultural technology innovation and adoption worldwide. Moreover, the U.S. is a pioneer and global leader in sweet corn research, development, and seed supply. Most sweet corn is grown for processing with a majority produced in the Midwest and the Northwest^23^. Due to inherent differences across the vast spatial domain, climate change trends can have conflicting effects on crop production^24–26^. Therefore, accuracy in estimating the effect of climate change on crop yield improves with data resolution. For instance, field-level data is more valuable than at the county or state level.

Accessing and analyzing historic crop production data is useful to developing future mitigation and adaptation strategies in response to climate change. We utilized sweet corn data from 16,040 fields, recorded over 27 years and across diverse environments, to study the impacts of climate change trends on sweet corn. The objectives of this study were to:

1. Estimate yield sensitivities to changes in growing season temperature and total precipitation,
2. Estimate critical thresholds in non-linear temperature effects on sweet corn yield across diverse environments, and
3. Quantify yield losses from surpassing the upper temperature threshold during anthesis in sweet corn

## Methods

### Crop yield data and sites

Field-level sweet corn yield data were obtained from multiple U.S. vegetable processors from 1992 to 2018. This data was collected from growers’ contract fields (hereafter called ‘fields’) in the Midwest (*the states of IL, MN, WI*) and the Northwest (*WA*). The Midwest fields represent both rainfed and irrigated production regimes while the Northwest was entirely irrigated. All fields were categorized into three distinct production regions, i.e., Midwest-Irrigated, Midwest-Rainfed, and Northwest-Irrigated, based on water supply and geographic location (see Supplementary Fig. S1 online). Sweet corn production in the Midwest is largely under rainfed production systems, while crop water needs are supplemented by irrigation in sandy soils of Wisconsin. Washington is the leading producer of processing sweet corn in the Northwest with almost all of the sweet corn production concentrated in Yakima Valley and around the Columbia basin. The Yakima Valley receives most of its water supply from the melting snowpack and has a mediterranean climate characterized by hot, dry summers and cool, wet winters. The Materials Transfer Agreement governing the use of this dataset dictates strict confidentiality, including names of processors and contract growers.

### Climate data

Daily minimum and maximum air temperature, and precipitation data at 1/8$^\circ$ latitude by longitude resolution were obtained from the Daymet database^27^ for the study years. Anomalies in precipitation and average air temperature for the growing season were calculated as deviations from the 30-year normal (long-term averages) for both the Midwest and the Northwest production regions (see Supplementary Fig. S2 online). Weather data was then subset to field-specific growing season, bracketed by the planting and harvest dates for each field. A subset of weather data for the anthesis period also was retained to quantify the effect of temperature during anthesis on sweet corn yields (see Supplementary Fig. S3 online). Anthesis period for each field was defined by the duration of ten days after the VT growth stage^28^.

### Data analysis

#### Estimation of yield sensitivities to precipitation and temperature

To understand the relationship between crop yield and weather variables (growing season total precipitation and mean temperature), we used the first-difference time series for yield and weather variables as described in^29^. By using the first-difference crop yield time-series, the influence of technological advancements in crop management and genetics are minimized. Multiple linear regression was performed with first differences in yield ($\Delta {\varvec{y}}{\varvec{i}}{\varvec{e}}{\varvec{l}}{\varvec{d}})$ as the response variable and first differences of growing season total precipitation $\left(\Delta {\varvec{P}}\right)$ and mean temperature $\left(\Delta {\varvec{T}}\right)$ as predictor variables for each production region.

The slope coefficients for both $$\Delta {\varvec{P}}$$ and $$\Delta {\varvec{T}}$$ were retained from each of the regression models. These slope coefficients represent yield increments per unit change (or sensitivity) in the growing season total precipitation (Mt ha^−1^ mm^−1^) and mean temperature (Mt ha^−1^$$^\circ{\rm C} $$^−1^). Production region–mean yields for the study period were calculated. The contribution of each of the weather variables to yield trends were represented in percent as the ratio of change in weather-attributed yield to the production region–mean yield. This was done to denote the proportion of changes in yield due to weather trends relative to the mean yield of a production region.

#### Regression model for relationship between temperature and crop yield

Following the methodology detailed in^30^, we calculated the total number of days spent in each 1 $^\circ{\rm C}$ temperature bin during growing season for each field. Daily hourly temperature values were estimated using a sine curve interpolation on daily minimum and maximum temperature.

#### The model

A panel regression was fitted at production region level following the approach given by^30^. The following equation was implemented in the regression model:

$${\varvec{l}}{\varvec{o}}{\varvec{g}}{{\varvec{Y}}}_{{\varvec{i}}{\varvec{t}}}={\boldsymbol{\alpha }}_{0}+\sum_{{\varvec{h}}=0}^{40}{{\varvec{\gamma}}}_{{\varvec{h}}}\left[{{\varvec{\theta}}}_{{\varvec{i}}{\varvec{t}}}\left({\varvec{h}}+1\right)-{{\varvec{\theta}}}_{{\varvec{i}}{\varvec{t}}}\left({\varvec{h}}\right)\right]+{{\varvec{z}}}_{{\varvec{i}}{\varvec{t}}}{\varvec{\delta}}+{{\varvec{c}}}_{{\varvec{i}}}+\boldsymbol{ }{{\varvec{\varepsilon}}}_{{\varvec{i}}{\varvec{t}}}$$

where ***Y*** is yield, ***i*** the field and ***t ***the year. ${{\varvec{\theta}}}_{{\varvec{i}}{\varvec{t}}}$ is the cumulative distribution of time (days) during the growing season spent at temperature ***h.*** ${\boldsymbol{\alpha }}_{0}$ represents a common intercept for all counties in a production region and ${{\varvec{c}}}_{{\varvec{i}}}$ is a county-specific fixed effect. ${{\varvec{z}}}_{{\varvec{i}}{\varvec{t}}}$ represents a matrix of exogenous variables like variability in precipitation (linear and quadratic), study year (linear and quadratic) to account for technological advancements that can influence crop yield with the fitted scaling factor ${\varvec{\delta}}.$ The spatially correlated residual errors are denoted by ${{\varvec{\varepsilon}}}_{{\varvec{i}}{\varvec{t}}}$. All temperatures above 40 $^\circ{\rm C}$ were pooled into 40 $^\circ{\rm C}$ temperature bin, while the temperatures below 0 $^\circ{\rm C}$ are captured by the fitted intercept.

#### Extreme heat stress during anthesis and crop yield

The effect of extreme heat stress during anthesis period on sweet corn yields was modelled using a linear mixed effects model shown below:

$${{\varvec{Y}}}_{{\varvec{i}}{\varvec{p}}{\varvec{t}}}={\varvec{a}}{{\varvec{X}}}_{{\varvec{i}}{\varvec{p}}{\varvec{t}}}+{{\varvec{b}}}_{{\varvec{p}}}+{{\varvec{c}}}_{{\varvec{t}}}+{{\varvec{\varepsilon}}}_{{\varvec{i}}{\varvec{p}}{\varvec{t}}}$$

where ${{\varvec{Y}}}_{{\varvec{i}}{\varvec{p}}{\varvec{t}}}$ is the natural logarithm transformed crop yield at field ***i*** in production region ***p*** and year ***t***, ${{\varvec{X}}}_{{\varvec{i}}{\varvec{p}}{\varvec{t}}}$ is a vector of weather variables for that field, ${\varvec{a}}$ is a vector of slope coefficients, ${{\varvec{b}}}_{{\varvec{p}}}$ represents an intercept associated with production region ***p***, ${{\varvec{c}}}_{{\varvec{t}}}$ denotes an intercept for study year ***t*** and ${{\varvec{\varepsilon}}}_{{\varvec{i}}{\varvec{p}}{\varvec{t}}}$ is the error term. The weather variables in ${{\varvec{X}}}_{{\varvec{i}}{\varvec{p}}{\varvec{t}}}$ included: GDD_8,30_, the sum of growing degree days between 8 and 30 $^\circ{\rm C}$ for the field-specific growing season, EDD_30+_, Extreme Degree Days, the sum of degree days above 30 $^\circ{\rm C}$, and total precipitation for the 10-day field-specific anthesis period. GDD_8,30_ is a commonly used metric to predict crop growth and development while EDD_30+_ is a measure of crop exposure to threshold temperatures that can result in reproductive failure in sweet corn due to extreme heat stress.

## Results

**Figure 1:**
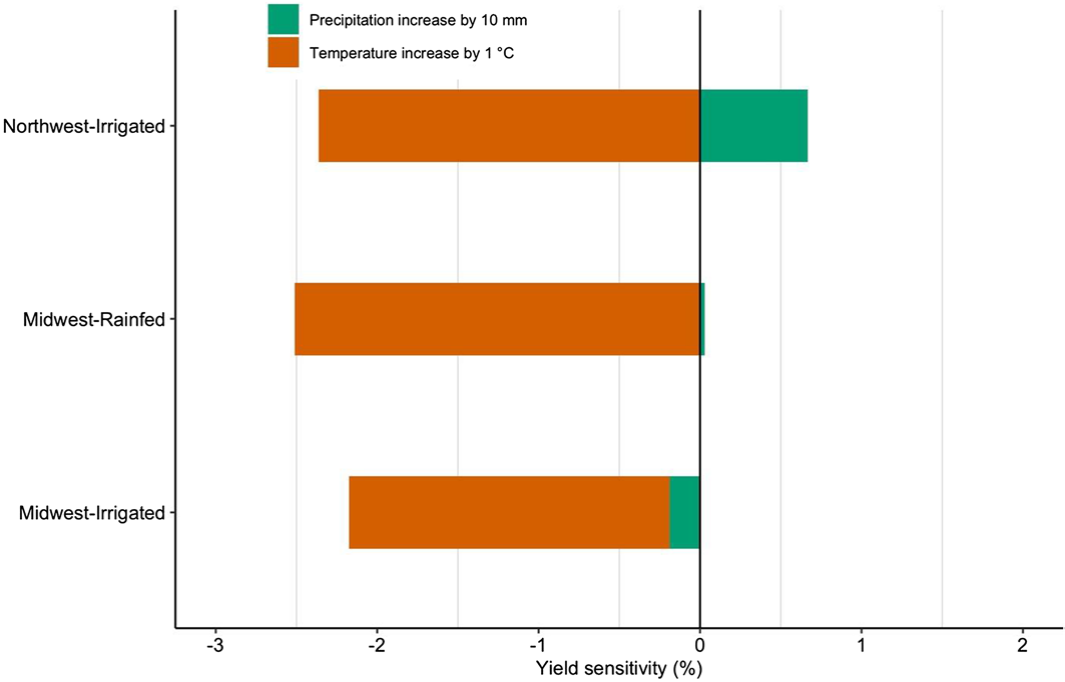
Sweet corn yield sensitivity to changes in growing season average temperature and total precipitation across the Midwest (Irrigated and Rainfed) and the Northwest (Irrigated) using commercial crop production data from 1992 to 2018. Yield sensitivities were estimated as percent yield losses relative to the mean yield for each production region.

We estimated the impacts of precipitation and temperature on crop yield across the spatial and temporal gradients of U.S. sweet corn production. Both magnitude and direction of changes in crop yield are shown (Fig. 1). Our analysis reveals that unit change in temperature has a greater influence on crop yield compared to precipitation changes for every production region. Overall, a unit increase in growing season mean temperature results in crop yield losses; however, the rainfed production region shows greater sensitivities compared to irrigated production regions. Furthermore, irrigated production of the Northwest showed a positive effect of increase in total precipitation, while Midwest-Irrigated reported negative effect of increase in total precipitation.

**Figure 2:**
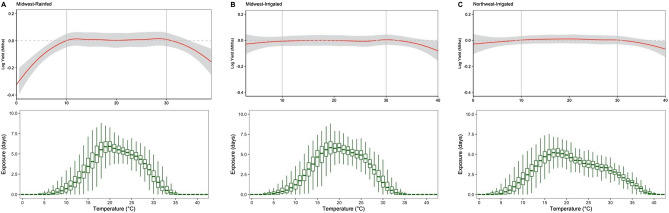
Nonlinear relation between temperature and yield for the (**A**) Midwest-Rainfed, (**B**) Midwest-Irrigated, and (**C**) Northwest-Irrigated. Graphs at the top of each frame display changes in log yield if the crop is exposed for one day (24 h) to a particular 1 °C temperature interval. Exposure (days) was calculated as the sum of the fraction of a day during which temperatures fall within each interval. The 95% confidence intervals are shown by gray shaded area. Histograms at the bottom of each frame display the average temperature exposure for all yield observations in each production region.

Our model estimates the changes in crop yield if the crop is exposed to temperatures in each unit Celsius temperature bin for a day (Fig. 2). A value of $\gamma$ = − 0.2 corresponding to temperature bin 40 $^\circ{\rm C}$ for Midwest-Rainfed production region means that one additional day at this temperature can reduce crop yields by 20%. Our models provide supporting evidence for no yield losses in the temperature range of 8 $^\circ{\rm C}$ yield loss. The statistical model shows rapid yield decline at temperatures exceeding $30^\circ{\rm C}$ in rainfed compared to irrigated production regions of the Midwest and Northwest (Fig. 2). This suggests that irrigation minimizes the negative effect of temperatures exceeding $30^\circ{\rm C}$ on crop yields; however, the negative effect of temperatures exceeding $35^\circ{\rm C}$ are visible in irrigated production regions as well.

**Figure 3:**
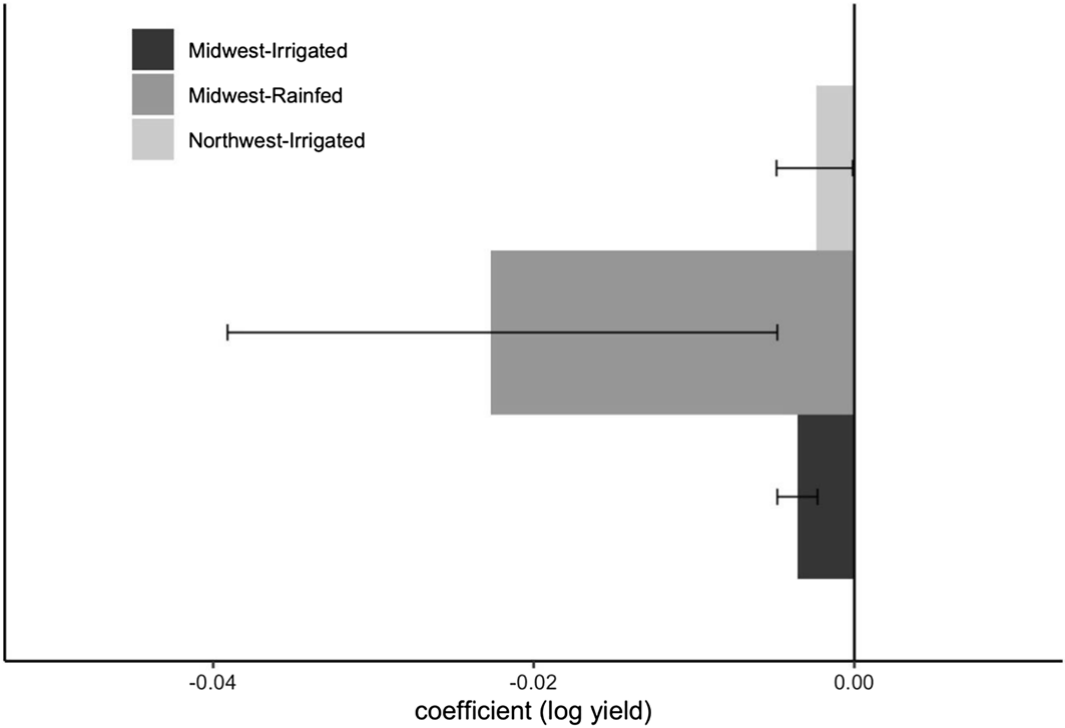
Regression coefficient estimates for the effects of extreme degree days (EDDs) by 1 degree day during anthesis on sweet corn yield (log scale) across the Midwest (Irrigated and Rainfed) and the Northwest (Irrigated) using commercial crop production data from 1992 to 2018. Extreme degree days were calculated using a base temperature of 30 $^\circ{\rm C}$ and no upper threshold for temperature. Error bars indicate bootstrapped 95% confidence interval using 1000 iterations.

Further effects of temperatures exceeding $30^\circ{\rm C}$ were quantified using the extreme degree day metric which was calculated using field-specific anthesis period hourly temperatures. We found a significant effect of EDDs on sweet corn yield; evidently higher yield losses for rainfed fields compared to irrigated fields (Fig. 3). A coefficient of − 0.02 (or − 0.005) can be interpreted as each additional degree day spent above $30^\circ{\rm C}$ reduces crop yield by 2% under rainfed conditions (or 0.5% under irrigated conditions). This provides further evidence that irrigation can mitigate some of the negative effect of heat stress due to high temperatures (> $30^\circ{\rm C}$) during sweet corn anthesis.

## Discussion

This study investigates historic sweet corn data in the context of climate change to unravel the significance of local weather anomalies on sweet corn yield across diverse environments. To our knowledge, this is the first use of a multi-year, data-intensive study that utilizes field-level sweet corn yield data to evaluate and quantify crop response to climate change.

Sweet corn yield response to climate change—capturing both temperature and precipitation trends—showed substantial variability across the vast spatial domain that encompasses commercial vegetable production. Recent trends towards increasing temperatures are detrimental to sweet corn yield, even with irrigation. Coupled with growing evidence of increases in frequency and intensity of heatwaves^2^, this poses a serious threat to the U.S. vegetable industry as a significant number of hectares of sweet corn are grown under rainfed conditions^31^. A more varied response to changing precipitation was observed. The arid Northwest production region can benefit from greater precipitation, especially when future projections report a drastic decline in ‘snowpack’ for the Northwest^32^. The Midwest production region did not show a pronounced effect of greater precipitation on sweet corn yields. This could be partly because of more diverse growing environments and multiple growing seasons aggregated in the Midwest region.

Our analysis, using the whole distribution of growing season temperatures, showed that temperatures ranging from 8 to 30 $^\circ{\rm C}$ represent ‘benign’ growing conditions for sweet corn. Previous empirical studies conducted under controlled environmental conditions have determined the same range of temperatures beneficial for field corn growth and development^33–35^. This suggests that our statistical framework is robust and accurately captures the non-linearities in temperature effects. More importantly, our models capture the detrimental effects of crop exposure to temperatures exceeding 30 $^\circ{\rm C}$ on yields which are exacerbated in rainfed production and similar to the previous findings from^36^. This is worrisome as the recent trends toward colder, wetter Midwest spring weather^37^ often results in delayed spring planting^23^, thereby exposing the crop to hot summer weather for a longer duration.

A large body of evidence reports reproductive phases of several crops are more sensitive to temperature stress than vegetative growth^38,39^. Furthermore, frequent occurrence of heat stress coinciding with flowering in certain crops is aggravating yield loss^40–42^. Our results confirm that high temperatures (above 30 $^\circ{\rm C}$) during anthesis reduced sweet corn yields across the U.S., with higher losses in rainfed environments. Each additional degree day spent above 30 $^\circ{\rm C}$ during anthesis reduced green ear yield by 2% in rainfed production. This is alarming as the future trends project 20–30 more days over 32 $^\circ{\rm C}$ than current in much of the U.S. by mid-century^37^. Furthermore, heat stress during grain filling decreases the activities of sucrose phosphate synthase (SPS) and sucrose synthase (SuSy), resulting in decreased sucrose content; consequently, deteriorating sweet corn eating quality^43^.

As the effects of climate change become widespread and pronounced it is imperative to implement a prioritized strategy to accelerate crop adaptation strategies to climate change^2^. A holistic approach involving germplasm improvement, site-specific agronomic management, climate forecasting, and real-time insights and predictive modeling could be explored to build climate resilient crop production systems. The cooling effect of irrigation has been documented to mitigate heat stress in some cereal cropping systems^44–46^; however, feasibility of irrigation is limited. Therefore, a cross-cutting interdisciplinary scientific approach that transcends across disciplines is required to address complex challenges posed by climate change.

In conclusion, we successfully quantified the impacts of spatial and temporal gradients of temperature and precipitation on sweet corn yield. Recent trends of increasing temperatures result in yield losses across the spatial domain. The effect of precipitation anomalies varied by production region. High temperatures (> 30 $^\circ{\rm C}$) during anthesis in sweet corn resulted in significant yield losses which were exacerbated under rainfed conditions. Producing sweet corn, one of the most popular vegetable crops in the U.S., will be more difficult in the future without adopting new approaches and technologies for crop adaptation to climate change.

## Supplementary Information

Supplementary Figures.

## Data Availability

The data that support the findings of this study are available from multiple U.S. vegetable processors but restrictions apply to the availability of these data, which were used under license for the current study, and so are not publicly available. Data are however available from the corresponding author upon reasonable request and with permission of the multiple U.S. vegetable processors.
