# Supplementary material for: Evidence of sweet corn yield losses from rising temperatures
Source: Sci Rep. 2022 Oct 29;12:18218. doi: 10.1038/s41598-022-23237-2 (PMC9617927; doi:10.1038/s41598-022-23237-2)

**Supplementary Material**

# Evidence of sweet corn yield losses from rising temperatures

**Daljeet S. Dhaliwal[1], Martin M. Williams Ⅱ[2*]**

[1]Department of Crop Sciences, University of Illinois at Urbana Champaign, Urbana, IL, United States

[2]Global Change and Photosynthesis Research Unit, USDA-ARS, Urbana, IL, United States

[*]**Author for correspondence:** Martin M. Williams Ⅱ

Email: martin.williams@usda.gov

**Supplementary Fig S1 County-level state maps for location of sweet corn fields included in the study.** Contract grower fields were in the Midwest (IL, MN, WI) and the Northwest (WA) regions. Counties under irrigated and rainfed production systems are shaded in blue and orange colors, respectively. Contract growers included in the study were present only in the shaded (blue or orange) counties

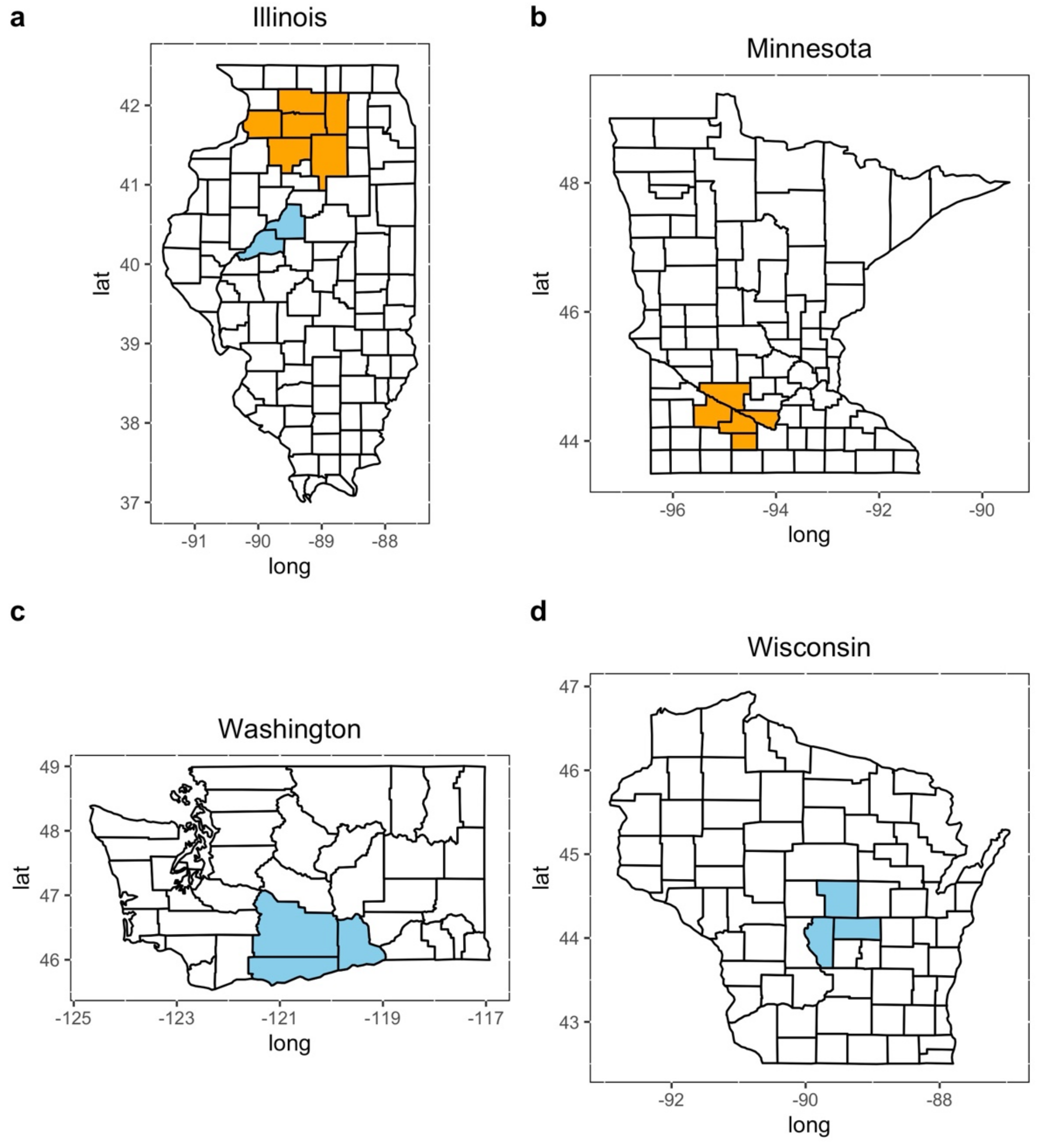

**Supplementary Fig S2 Weather anomalies in growing season total precipitation and average air temperature for (a) the Midwest, and (b) the Northwest production regions.** Anomalies (%) were calculated as deviations from the 30-yr normal for the precipitation and air temperature during the months of sweet corn growing season (April-September) for the respective regions. Study years showing significant weather anomalies are labelled.

**(a) The Midwest**

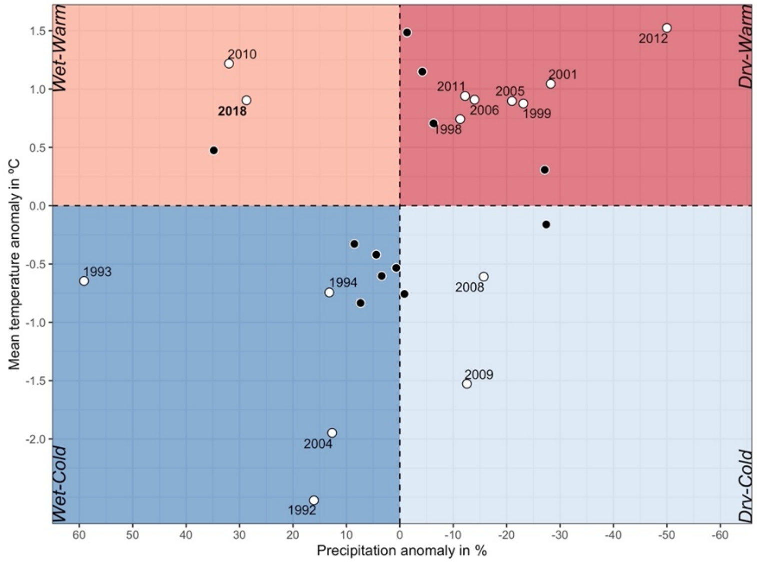


**(b) The Northwest**

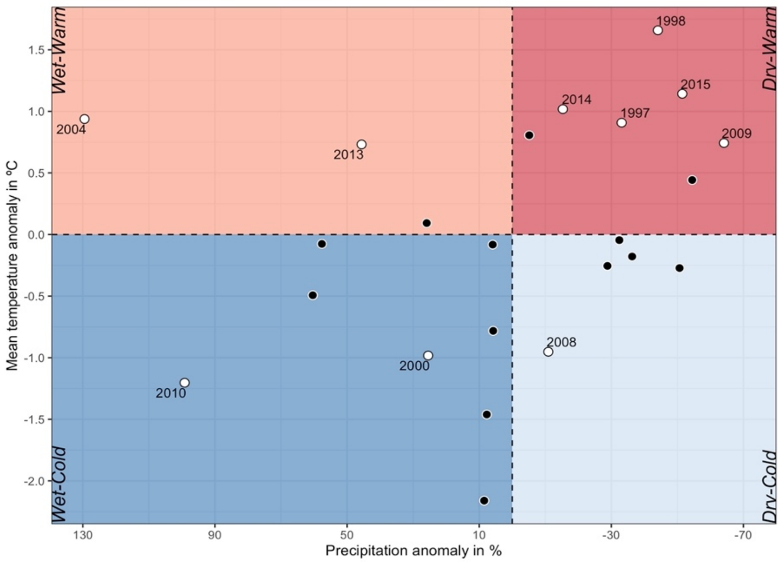

**Supplementary Fig S3 Scatterplots for relationship between air temperatures (maximum and average air temperatures during sweet corn anthesis) and sweet corn yield.** Panels (a-b), (c-d), and (e-f) show the scatterplots for regions Midwest-rainfed, Midwest-irrigated, and Northwest-irrigated, respectively.

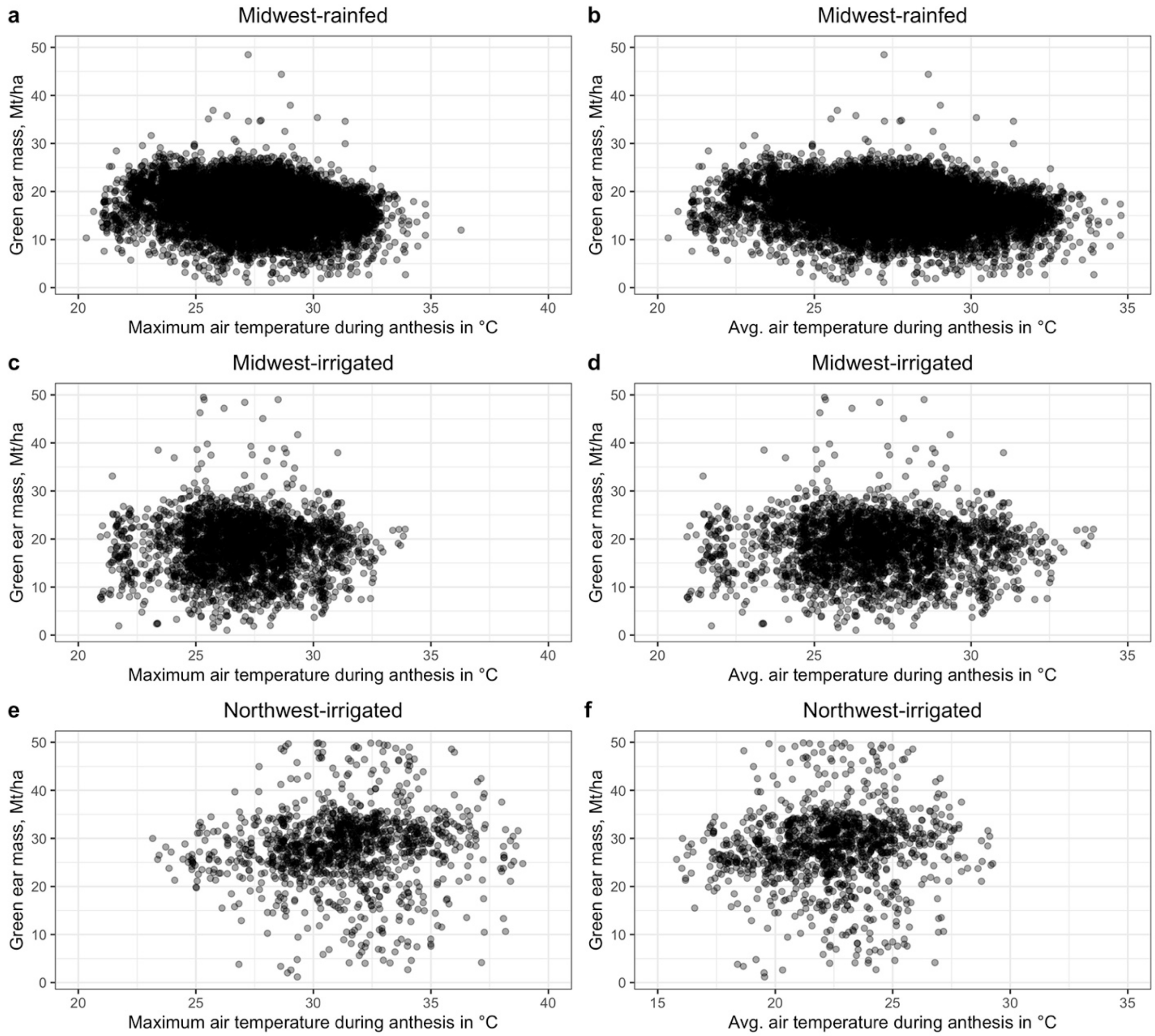

Supplement: Supplementary file 1 — Supplementary Figures. [file 41598_2022_23237_MOESM1_ESM.pdf]
